# A case report of a Chinese patient with obstructive hypertrophic cardiomyopathy harboring rare variants in both the MYBPC3 and DSP genes

**DOI:** 10.3389/fcvm.2025.1630263

**Published:** 2026-01-05

**Authors:** Xiao-Yuan Wu, Ning Ren, Jie Geng

**Affiliations:** Department of Cardiology, Chest Hospital, Tianjin key Laboratory of Cardiovascular Emergency and Critical Care, Tianjin Municipal Science and Technology Bureau, Tianjin University, Tianjin, China

**Keywords:** cardiac myosin-binding protein C gene variant, case report, desmoplakin gene variant, hypertrophic cardiomyopathy, rare variants

## Abstract

Hypertrophic cardiomyopathy (HCM) represents the most prevalent form of hereditary cardiomyopathy, and mutation in the cardiac myosin-binding protein C (MYBPC3) gene have been identified as a major contributor to the pathogenesis of HCM. While the desmoplakin (DSP) gene is primarily associated with arrhythmogenic right ventricular cardiomyopathy (ARVC) and dilated cardiomyopathy (DCM), its role in HCM has been less frequently documented. This case report describes a Chinese patient with obstructive HCM harboring rare variants in both the MYBPC3 and DSP genes. These findings provide valuable insights for future investigations into the genetic underpinnings and disease associations.

## 1 Introduction

Hypertrophic cardiomyopathy (HCM) is a primary myocardial disorder characterized by unexplained left ventricular hypertrophy. It is primarily caused by pathogenic variants in sarcomere-related genes or classified as idiopathic after excluding secondary causes like hypertension or valvular disease (1). While over twenty genes have been implicated in HCM, the majority of genetically confirmed cases arise from variants in two core sarcomeric genes: the β-myosin heavy chain 7 gene and the cardiac myosin-binding protein C (MYBPC3) gene, collectively accounting for 50%–60% of cases (2). In contrast, the desmoplakin (DSP) gene, encoding the primary component of desmosomes, has been predominantly associated with arrhythmogenic right ventricular cardiomyopathy (ARVC) and dilated cardiomyopathy (DCM) (3, 4), with limited evidence supporting its role in HCM pathogenesis. Most individuals with heterozygous MYBPC3 variants in HCM typically have a late age of onset and a benign clinical course (5). However, certain pathogenic variants are linked to more malignant phenotypes, which can lead to rapid progression to heart failure and, in severe cases, necessitate heart transplantation. Thus, early genetic testing is vital for accurate risk stratification and long-term prognostic assessment in HCM (6).

Here, we present a case of a patient with resting obstructive HCM, harboring two rare variants in the MYBPC3 and DSP genes.

## 2 Case presentation

The proband was a 40-year-old male who presented with exertional dyspnea for one year and substernal chest pain for one week. He had no history of hypertension, diabetes mellitus, or dyslipidemia and denies any history of smoking or alcohol consumption. His father experienced sudden cardiac death (SCD) at the age of 41; however, no specific details regarding his premorbid symptoms or postmortem findings are available.

**Figure 1:**
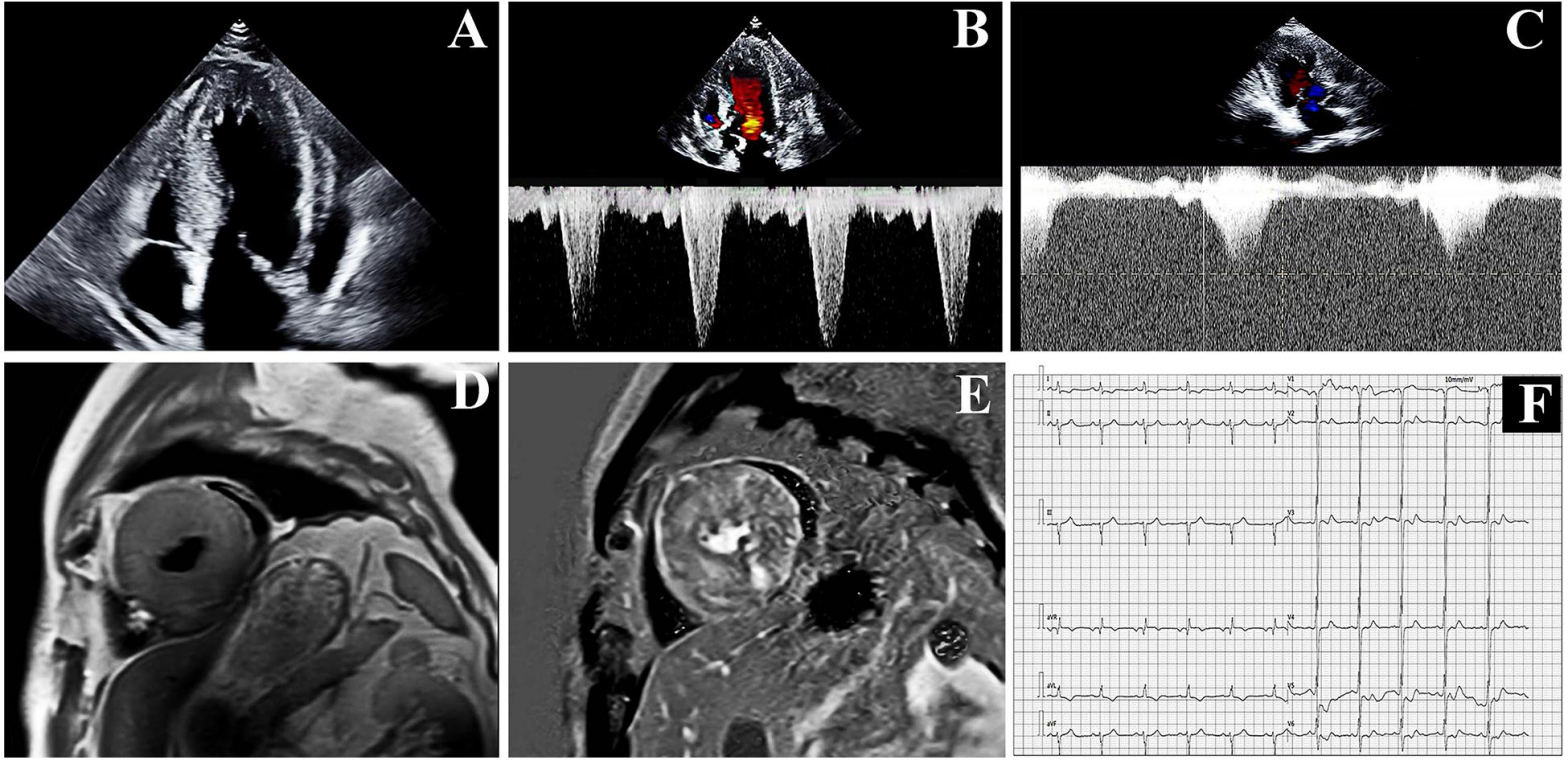
The proband's examination results: **(A,B)** echocardiographic results at hospitalization. **(C)** Echocardiographic results at the 1-month follow-up. **(D,E)** Cardiac Magnetic Resonance (CMR) at hospitalization. **(F)** Electrocardiogram result.

On admission, vital signs showed blood pressure 111/61 mmHg and a heart rate 60 bpm. Cardiac auscultation revealed a grade 3/6 harsh systolic ejection murmur at the left sternal border (3rd–4th intercostal spaces), accentuated by Valsalva maneuver. Echocardiography revealed the following key findings: severe asymmetric septal hypertrophy, characterized by an interventricular septal thickness (IVsd) at end-diastole of 25–30 mm and a left ventricular posterior wall dimensions (LVPWd) of 21 mm, with a preserved left ventricular ejection fraction (LVEF) of 59% (Figure 1A). A dynamic left ventricular outflow tract (LVOT) obstruction: systolic anterior motion (SAM) of the mitral valve, resting LVOT velocity of 4.65 m/s, and peak LVOT gradient of 86 mmHg (normal <30 mmHg) (Figure 1B). Laboratory findings revealed markedly elevated TSH (>100.00 μIU/mL; normal range: 0.4–4.0 μIU/mL) with reduced free T4 (1.51 pmol/L; normal range: 12.8–21.3 pmol/L). Hyperlipidemia was noted with low-density lipoprotein cholesterol (LDL-C) of 5.65 mmol/L. Additionally, there were elevated levels of B-type natriuretic peptide (BNP) (449.64 pg/mL; normal range <100 pg/mL), creatine kinase (CK) (503 U/L; normal range<190 U/L), and high-sensitivity cardiac troponin T (hs-cTnT) (0.132 ng/mL; normal range: <0.014 ng/mL).

Cardiac Magnetic Resonance (CMR) demonstrated diffuse hypertrophy of all left ventricular segments (at the papillary muscle level: interventricular septum 24–25 mm, anterior wall 21–22 mm, inferior wall 26–27 mm, lateral wall 21–22 mm) and extensive myocardial fibrosis. T1 mapping values were 1,456 ± 91 ms, and extracellular volume fraction was 38% ± 6% (Figures 1D,E). Systolic and diastolic functions of the left ventricle were impaired, with increased trabeculation indicative of excessive left myocardial trabeculation. Electrocardiography (EKG) showed sinus rhythm at 67 bpm with left anterior fascicular block (LAFB) and deep T-wave inversions in leads I, aVL, and V2 to V6. No ST-segment deviations or pathological Q waves (Figure 1F). The 24-hour Holter EKG revealed a total of 94,738 heartbeats, characterized by sinus arrhythmia, sporadic atrial premature contractions, sporadic ventricular premature contractions with occasional bigeminy, and ST-T segment changes. Contrast-enhanced cardiac CT revealed a mild stenosis of approximately 30% in the left anterior descending artery (LAD).

**Figure 2:**
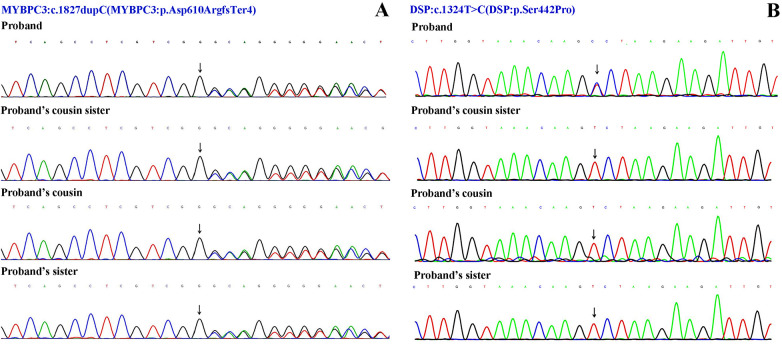
Sanger sequencing results. **(A)** The variant NM_000256.3: c.1827dupC (p. Asp610ArgfsTer4) of MYBPC3 gene. **(B)** The variant NM_004415.4: c.1324T>C (p. Ser442Pro) of DSP gene.

**Figure 3:**
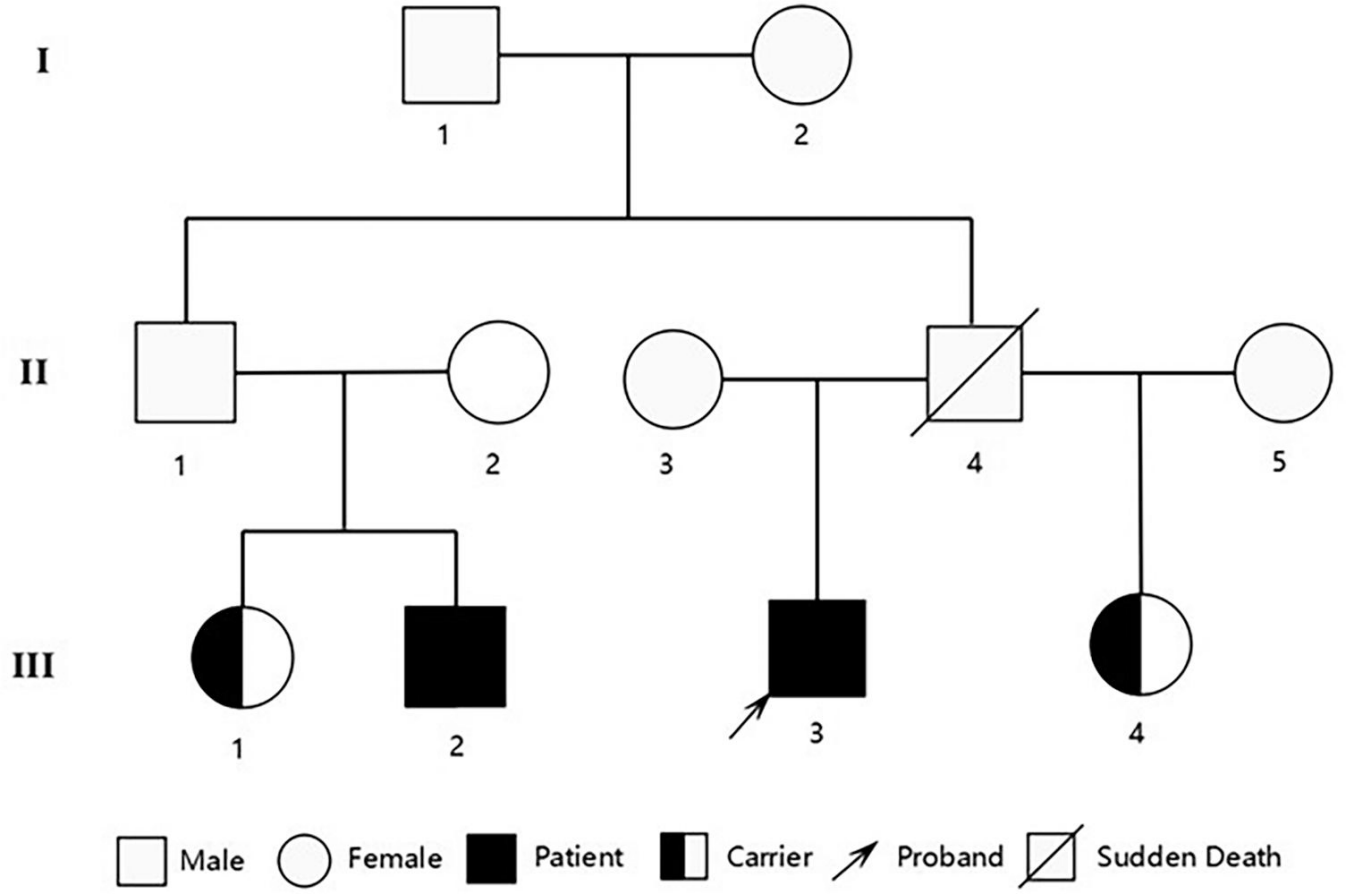
Pedigree of the family showing the genotypes of the proband and affected relatives. Male family members are represented by a square and female family members are represented by a circle.

Although severe hypothyroidism can impair systolic and diastolic function and induce myocardial edema (which may mimic hypertrophy), the patient's CMR showed no evidence of diffuse edema (7). The late gadolinium enhancement (LGE) pattern was instead consistent with the fibrosis characteristic of HCM. Moreover, the findings of asymmetric septal hypertrophy and LVOT obstruction—not typically associated with thyrotoxic heart disease—further support a primary diagnosis of HCM. According to the 2024 AHA/ACC/AMSSM/HRS/PACES/SCMR Guideline for the Management of Hypertrophic Cardiomyopathy (1), the proband was diagnosed with resting obstructive HCM. To rule out other metabolic and infiltrative disorders, including cardiac amyloidosis and Fabry disease, a comprehensive evaluation was performed. The serum free light chain (FLC) assay showed no abnormalities, and 99mTc-PYP scintigraphy revealed no myocardial tracer uptake, effectively excluding transthyretin-related cardiac amyloidosis (ATTR-CM) and rendering light-chain amyloidosis (AL-CM) highly unlikely. Furthermore, both alpha-galactosidase A enzyme activity and GLA gene sequencing yielded normal results, definitively ruling out Fabry disease. Finally, despite a history of hyperlipidemia, CMR imaging demonstrated no evidence of myocardial fat infiltration, thereby arguing against a diagnosis of lipotoxic cardiomyopathy. After obtaining ethical approval and written informed consent, target-region capture sequencing and second-generation high throughput sequencing were performed to analyze all exons and their flanking regions. Rare and known variants were validated using Sanger sequencing and cascade screening was carried out for all available relatives. The classification of genetic variants was determined in accordance with the most recent international medical consensus. The genetic analysis identified a rare heterozygous variant NM_000256.3: c.1827dupC (p. Asp610ArgfsTer4), in exon 18 of the MYBPC3 gene and a novel heterozygous missense variant NM_004415.4: c.1324T>C (p. Ser442Pro), in exon 11 of the DSP gene. The proband's father had a history of SCD at 41 years of age. His mother declined genetic testing, and his children were too young to undergo such testing. Targeted region capture and second-generation high throughput sequencing were performed on the proband's cousin, cousin sister, and sister. Results demonstrated that all family members carried the MYBPC3 c.1827dupC variant, whereas only the proband carried the DSP c.1324T>C variant; the other family members did not carry this mutation (Figures 2, 3).

The proband carried a heterozygous MYBPC3 variant (NM_000256.3: p. Asp610ArgfsTer4). This duplication caused the substitution of aspartic acid, a negatively charged polar amino acid at position 610, with arginine, a positively charged polar amino acid, resulting in a frameshift variant and the incorporation of a premature stop codon three residues downstream. Population databases revealed this variant to be rare (1000 Genomes: none, ESP6500: none; ExAC: none). No records of this variant were identified in the ClinVar database. However, according to the Human Gene Mutation Database (HGMD), this variant was classified as a “disease-causing mutation”. A search of the ClinVar database revealed that frameshift or nonsense variants downstream of this locus have been consistently classified by submitters as pathogenic or likely pathogenic for HCM. According to the American College of Medical Genetics (ACMG) guidelines (8), this variant is categorized as likely pathogenic, meeting the following criteria: PVS1 + PM2.

Simultaneously, the proband was found to carry a heterozygous missense variant c.1327T>C in the DSP gene (DSP: p. Ser442Pro). Population frequency databases revealed that this variant is extremely rare (1000 Genomes: none, ESP6500: none, ExAC: none). A search of the ClinVar and HGMD databases did not identify this specific variant; however, a nearby missense variant, c.1351C>G (p. Arg451Gly), was classified as pathogenic in ClinVar database, although the associated disease was not specified. The substitution of a polar and uncharged serine with a nonpolar proline at this position alters amino acid properties and may impact protein function. Bioinformatics analysis revealed that this residue is highly conserved across vertebrates. To assess the functional impact of the missense variant c.1327T>C in the DSP gene, we employed multiple bioinformatics prediction tools, including SIFT, PolyPhen-2, and others. The results indicated that this variant was predominantly predicted to be deleterious (SIFT: “D”, PolyPhen-2: “D”, Mutation Taster-pred: “D”, WEST score: 0.326; other predictions included 4 “D”s, 3 “T”s, and 1 “M”). According to the guidelines of ACMG, while this variant is currently classified as a variant of uncertain significance (VUS), it satisfies two criteria for likely pathogenicity: PM2 + PP3. Based on the available evidence, this variant is rare and is predicted to potentially impact protein function.

Echocardiographic evaluations were performed on the family members. The results revealed that the cousin exhibited an IVSd of 13 mm, a LVPWd of 11 mm, and a LVEF of 59%. Based on these findings and his clinical characteristics, the cousin was also diagnosed with HCM (1). Additionally, the echocardiographic parameters of the other two family members were all within normal limits.

One month after thyroid function and serum lipid levels normalized through gradual dose uptitration of levothyroxine, repeat echocardiography revealed persistent severe cardiac abnormalities. The echocardiogram indicated that the LVEF was 57%. Although the thickness of the ventricular wall remained unchanged, the flow velocity in the LVOT slowed down to 4.3 m/s, and the pressure gradient in the LVOT decreased to 75 mmHg (Figure 1C). BNP levels decreased to 399.52 pg/mL.

## 3 Discussion

The MYBPC3 gene is located on the short arm of chromosome 11 at cytogenetic band 11p11.2, spanning approximately 21 kb of genomic DNA and containing a coding sequence of 4,217 nucleotides distributed across 35 exons. This gene encodes cardiac myosin-binding protein C (cMyBP-C), a sarcomeric scaffolding protein that is critical for both the structural integrity and functional regulation of cardiac muscle (9). The MYBPC3 gene is the most frequently mutated gene in HCM. To date, more than 800 pathogenic MYBPC3 variants related to HCM have been identified, with over half being truncating variants, including nonsense variants, insertions/deletions or splice site variants. The HCM phenotype associated with MYBPC3 truncating variants is more severe compared to missense and in-frame deletions (10, 11). Clinical manifestations of hypertrophic cardiomyopathy (HCM) associated with MYBPC3 gene variants typically emerge between the ages of 30 and 40. Notably, male sex and the presence of electrocardiogram abnormalities are correlated with a higher incidence of HCM (12, 13).

The proband carried MYBPC3: c.1827dupC (p. Asp610ArgfsTer4) variant. This may trigger nonsense-mediated mRNA decay leading to incomplete expression of cMyBP-C, encoded by the MYBPC3 gene. The resulting deficiency impairs its role in anchoring thick filaments, subsequently causing structural and functional abnormalities in sarcomeres, which may contribute to the development of HCM (14). In a sequencing study targeting eight genes, including MYBPC3, conducted among 529 unrelated HCM patients, one patient was found to carry this variant. However, the detailed clinical manifestations and characteristics of this patient were not explicitly reported (15). Our case report further delineates the HCM phenotype associated with MYBPC3: c.1827dupC variant. Through genetic evidence and echocardiographic findings, we successfully established an early diagnosis of HCM in the proband's cousin. Although two sisters currently exhibit preserved cardiac morphology, serial imaging surveillance is mandated per AHA/ACC guidelines to monitor subclinical remodeling.

The DSP gene is located at 6p24.3 on the short arm of chromosome 6 and consists of 24 exons. It encodes desmoplakin, which is the most abundant component in desmosomes and plays a critical role in cardiomyocyte junctions (16). Pathogenic variants in the DSP gene represent the most penetrant genetic drivers of arrhythmogenic cardiomyopathy, a mechanically uncoupled cardiomyopathy characterized by biventricular transmural fibroadipocytic replacement (17). Patients with DCM linked to DSP variants exhibit a significantly increased risk of SCD and life-threatening ventricular arrhythmias, even when LVEF is only mildly reduced (18). DSP cardiomyopathy is pathologically defined by the early development of left ventricular fibrogenesis during subclinical stages, which precedes detectable systolic impairment. This pathological process is coupled with a pronounced propensity for malignant ventricular arrhythmias (19).

Most patients with MYBPC3-related HCM present with a later age of onset and less severe myocardial hypertrophy (5). Each MYBPC3 variant associated with HCM is linked to distinct clinical manifestations. This variability is not limited to patients with different pathogenic mutations but also extends to family members who carry the same mutation. Evidence has shown that monozygotic twins harboring identical MYBPC3 variants can exhibit divergent phenotypic manifestations and variable disease severity. This observation further suggests the potential contribution of unidentified modifying factors to the development and expression of HCM (20). Although the proband carries the same MYBPC3 variant as other family members, they exhibit a more severe clinical phenotype and an elevated risk of SCD. This observation further supports the possibility that the rare heterozygous missense variant c.1327T>C in the DSP gene may act as a genetic modifier by enhancing myocardial fibrosis and disrupting electrical conduction. However, the underlying pathogenic mechanisms remain to be fully elucidated.

Current evidence supporting the causal relationship between DSP gene mutations and HCM remains limited, with only sparse robust clinical data substantiating this association. Preclinical studies using mouse models with DSP variants have revealed significant disparities in the progression of exercise-induced ARVC. Specifically, at 16 weeks of age, mice harboring DSP variants consistently exhibited pathological ventricular septal hypertrophy, irrespective of exposure to long-term exercise stimulation. Additionally, the ratio of cardiac mass to body weight was significantly elevated (21). Pathogenic variants in the DSP gene may exacerbate the clinical phenotype of patients with HCM. A large-scale clinical study has demonstrated that patients with HCM carrying pathogenic DSP variants exhibit a distinct arrhythmia phenotype, characterized by non-sustained ventricular tachycardia (NSVT), left bundle branch block (LBBB), and biventricular structural remodeling (22).

SCD, heart failure (HF), and thromboembolic events represent the three primary causes of mortality in patients with HCM (23). Extensive late gadolinium enhancement (LGE), defined as involving ≥15% of the left ventricular myocardial mass index (LVMI), represents a critical prognostic indicator for risk stratification of SCD in HCM (24). In this case report, the proband carried two rare variants in genes associated with inherited cardiomyopathies and exhibited multiple high-risk features for SCD, including: (1) a family history of SCD, (2) severe left ventricular hypertrophy (maximal wall thickness ≥30 mm), (3) extensive LGE on CMR imaging, and (4) significant LVOT obstruction. Meanwhile, the proband presented with heart failure with preserved ejection fraction (HFpEF) characterized by diastolic dysfunction of the ventricles. Collectively, this evidence suggests an adverse prognosis for this patient. Although no documented malignant ventricular arrhythmias have been observed on electrocardiography to date, the proband was found to carry DSP: c.1324T>C (p. Ser442Pro) variant. Mechanistically, this variant may exacerbate myocardial fibrotic remodeling and impair electroconductive properties, potentially accelerating ventricular fibrosis progression and increasing susceptibility to life-threatening arrhythmias (22). The proband currently exhibits no severe ventricular arrhythmias and manifests only lAFB, which may suggest that the disease is in its very early stage. The LAFB in the proband may be attributable to the DSP variant affecting the specific conduction pathway of the left anterior fascicle. Although no ventricular arrhythmias have been documented to date, the risk of ventricular tachycardia is likely to increase as myocardial fibrosis progresses; therefore, long-term monitoring via continuous electrocardiographic recording is warranted.

However, the lack of familial segregation data and direct functional evidence limits the interpretation of these findings.

Although guideline-directed therapy for the primary prevention of sudden cardiac death—based on the 2024 AHA/ACC guidelines and the ESC HCM Risk-SCD model—supports ICD implantation, the decision to defer was made following multidisciplinary evaluation and shared decision-making with the patient and family. This decision took into account the patient's age, the recent diagnosis of HCM, and personal concerns regarding lifelong device management. Current management focuses on pharmacotherapy with beta-blockers and mavacamten to control heart rate and mitigate myocardial hypertrophy. The patient is enrolled in a rigorous surveillance protocol involving quarterly Holter monitoring and echocardiography to detect NSVT or progression of the LVOT pressure gradient. ICD implantation will be re-evaluated should high-risk features emerge, such as NSVT or a significant increase in LVOT obstruction.

## 4 Conclusion

This report presents a clinical case of resting obstructive hypertrophic cardiomyopathy with two rare novel genetic variants. Through phenotypic correlation and familial segregation analysis, we further characterized the heterozygous MYBPC3: c.1827dupC (p. Asp610ArgfsTer4) and successfully achieved early diagnosis of HCM within the family. Furthermore, the DSP gene variants may act as phenotypic modifiers in HCM by exacerbating myocardial dysfunction, thereby providing important insights into revealing the genetic basis and disease progression mechanism of HCM.

Similarly, this report has certain limitations. Given the limited sample size, the causal relationship between genotype and clinical phenotype requires further validation through additional clinical evidence and rigorous functional studies.

## Data Availability

The datasets presented in this study can be found in online repositories. The names of the repository/repositories and accession number(s) can be found in the article/Supplementary Material.
